# Does Time between Imaging Diagnosis and Initiation of Radiotherapy Impact Survival after Whole-Brain Radiotherapy for Brain Metastases?

**DOI:** 10.1155/2013/214304

**Published:** 2013-04-11

**Authors:** Carsten Nieder, Oddvar Spanne, Ellinor Haukland, Astrid Dalhaug

**Affiliations:** ^1^Department of Oncology and Palliative Medicine, Nordland Hospital, 8092 Bodø, Norway; ^2^Institute of Clinical Medicine, Faculty of Health Sciences, University of Tromsø, 9037 Tromsø, Norway; ^3^Department of Oncology, University Hospital of Northern Norway, 9037 Tromsø, Norway

## Abstract

*Aims*. To evaluate whether reduced waiting time influences survival of patients treated with whole-brain radiotherapy (WBRT) for brain metastases. *Materials and Methods*. Retrospective intention-to-treat study including 110 patients treated with primary WBRT (typically 10 fractions of 3 Gy; no other treatment between diagnosis and WBRT). Uni- and multivariate tests were performed. *Results*. Median delay between imaging diagnosis and WBRT was 12 days (range 0–66 days). WBRT started within 1 week in 36%, during the second week in 28%, and during the third week in 18% of patients. No significant correlation between waiting time and survival was evident, except for one subgroup of patients. Those without extracranial metastases (potentially more threatened by worse intracranial disease control) survived for a median of 2.5 months from WBRT if waiting time was 2 weeks or longer as compared to 5.6 months if waiting time was shorter than 2 weeks (*P* = 0.03). The same correlation was seen if survival was computed from imaging diagnosis. *Conclusion*. If departmental resources are not sufficient to provide immediate WBRT within 2 weeks to all patients, those without extracranial metastases should be prioritised. This study did not address the impact of waiting time on quality of life or symptom palliation.

## 1. Introduction

On an international scale, access to palliative radiotherapy varies with geographic region, health care system, and sociodemographic factors [1, 2]. The presence of waiting lists might cause distress, unnecessary symptom burden, and under certain circumstances compromised outcomes, at least if long waiting time is unavoidable. Regarding treatment of brain metastases, a considerable number of patients continue to receive palliative whole-brain radiotherapy (WBRT) [3, 4]. Administration of 10 fractions of 3 Gy over 2 weeks or 5 fractions of 4 Gy over one week is commonly used fractionation regimens in many countries [5]. Given that median survival of patients managed with best supportive care is limited (in the order of 4–6 weeks [6, 7]), one might assume that delays in starting WBRT should be minimised, if such treatment is indicated and the preferred therapeutic option. Clinical data on the impact of variable waiting times between imaging diagnosis of brain metastases and initiation of WBRT on survival after radiotherapy are scarce [8]. Therefore, we evaluated survival of a contemporary cohort of patients treated with WBRT.

## 2. Patients and Methods

We analysed patients from a previously described multi-institutional brain metastases database, which is maintained and updated by the first author [7, 9]. The patients were treated at Nordland Hospital (Bodø) and University Hospital of Northern Norway (Tromsø). For this retrospective intention-to-treat study, all patients treated with primary WBRT (prescribed dose 10 fractions of 3 Gy or 5 fractions of 4 Gy; no previous surgery or radiosurgery; no radiation boost) between 2005 and 2012 were selected (*n* = 110). All patients who failed to complete WBRT are included in the analysis. The choice of WBRT dose was made by the treating physician taking into account the life expectancy of individual patients. WBRT commenced without preceding systemic therapy after imaging diagnosis of brain metastases. The patient characteristics are shown in Table 1. For comparison of dichotomous variables the Chi-Square Test and Fisher's Exact Test, where applicable, were employed and for continuous variables the Mann-Whitney *U* Test (always 2-sided). Actuarial survival was calculated with the Kaplan-Meier method and compared between different groups with the log-rank test. Thirteen patients (12%) were alive at date of the last followup (November 01, 2012) and thus censored. Their median followup was 5 months (range 1–59 months). The prognostic impact of baseline parameters and waiting time was first tested in univariate analyses (log-rank test). For multivariate analysis of survival Cox regression analysis was used. Waiting time was entered as continuous or categorical variable with different cutoff values. A *P* value <0.05 was considered statistically significant.

## 3. Results

Of all 110 patients included in the study 36 (33%) started WBRT within one week from imaging diagnosis of brain metastases. Another 28% (31 patients) started during the second week and 18% (20 patients) during the third week. Median delay was 12 days (range 0–66 days). Median survival was 3.0 months from imaging diagnosis and 2.5 months from the first dose of WBRT. As shown in Table 2, no significant correlation between waiting time and survival was evident. The small group of 16 patients (15%) with waiting time >28 days had median survival comparable to patients with waiting time ≤28 days (2.5 months from WBRT, *P* = 0.92, and 4.2 months from imaging diagnosis, *P* = 0.46). Given that most but not all baseline characteristics were balanced between the two groups with waiting time <12 days versus ≥12 days (as shown in Table 1 significant differences regarding primary tumour type were seen), a series of multivariate Cox regression analyses was performed. Waiting time was entered as categorical variable (stratified by median or 4 strata, i.e., <1 week, 1-2 weeks, 2-3 weeks, or more) or as continuous variable. Primary tumour type and prognostic scores (recursive partitioning analysis (RPA) classification [10] and diagnosis-specific graded prognostic assessment (DS-GPA) score [11]) were entered as covariates. No significant correlation between waiting time and survival from WBRT or imaging diagnosis was identified (details not shown). 

In order to explore the hypothesis that specific subgroups of patients could be more vulnerable to detrimental effects of longer waiting time, more detailed analyses were performed. Irrespective of cutoff chosen, Karnofsky performance status (KPS) and age did not turn out to be relevant. However, extracranial disease extent might be important. In patients with extracranial metastases, survival depends on both intra- and extracranial disease control and is typically shorter than in patients without extracranial metastases (3.0 versus 4.2 months in the present study). In patients with brain metastases only, intracranial disease control is more important, and delayed WBRT could threaten survival. Twenty-one patients in this study did not harbour extracranial metastases. Median survival was 2.5 months from WBRT with waiting time ≥2 weeks and 5.6 months with shorter waiting time (*P* = 0.03). Comparable results were seen for survival from imaging diagnosis (3.8 versus 5.9 months, *P* = 0.03). 

The risk of being unable to complete the prescribed course of WBRT was similar across all groups (9% each for interval <12 days versus ≥12 days, 9% for start within one week, 11% for start during the second week, 5% for start during the third week, 10% for start after more than 3 weeks, and *P* > 0.6 for both statistical approaches).

Survival from imaging diagnosis was significantly longer in patients with breast cancer (median 13.9 months) and small cell lung cancer (median 5.3 months) than those with other primary diagnoses. Both RPA classification and DS-GPA score also predicted survival. Median was 29.9 months in RPA class I, 4.2 months in class II, and 2.1 months in class III (*P* = 0.0001). Median was not reached for DS-GPA class I, 4.4 months for class II, 3.7 months for class III, and 2.3 months for class IV (*P* = 0.002). In the multivariate analyses mentioned above only RPA and primary tumour type remained statistically significant.

## 4. Discussion

Few if any patients with brain metastases would be willing to participate in a randomised trial examining the impact of different waiting times on outcomes after WBRT. In the absence of such trials, the present retrospective intention-to-treat analysis provides interesting results. As shown in Table 2, the data do not suggest that moderately delayed WBRT compromises survival for the majority of patients. Whether or not other endpoints such as neurological function status, steroid dependency or patient reported quality of life might be compromised is currently unknown and cannot be examined retrospectively. Given that extended waiting times are unlikely to provide any medical advantage, efforts should be undertaken to minimise delay between diagnosis and WBRT. Our data suggest that it might be justified to prioritise patients without extracranial metastases in cases where departmental waiting lists cannot be avoided, with the reason being that untreated brain metastases might shorten survival in patients who are not immediately threatened by progression in extracranial sites. However, it must be acknowledged that other parameters might also be important. Such parameters might include neurological function, symptom burden, mass effect, or intracranial tumour volume. Due to a lack of recording in our database we were unable to include any of them. Regarding further potential shortcomings of this study, one should be aware of its retrospective nature and the limited patient numbers.

The major reason for variable waiting times in the present patient population was limited capacity at the treating institutions, but the magnitude of this problem was not constant over time and impacted patients quite randomly. For each given year, seasonal variations, machine maintenance, and breakdown episodes occurred. Occasionally, delayed WBRT was caused by unexpected intercurrent comorbid conditions or patient request. Even during time periods with heavy workload and longer waiting lists, doctors could influence to some degree whether a given patient started WBRT rapidly because a certain number of emergency slots existed. If doctors are effective at distinguishing between more and less aggressive disease states, the impact of waiting time on outcomes might be diminished. These considerations might also explain why some studies on delay of postoperative radiotherapy for glioblastoma described a correlation between longer waiting time and decreased survival while others did not [12]. A combined analysis suggested that moderate wait periods (up to 4–6 weeks) are safe [13], but these data are difficult to compare with ours (unresected brain metastases).

In a previous study by Lutterbach et al. waiting time was examined only for patients who underwent biopsy or resection before WBRT [8]. Median delay was 13 days, but time from initial imaging diagnosis was not reported. Impact on survival was not analysed either. In a recent randomised trial of WBRT with or without motexafin gadolinium, the median time from brain metastasis diagnosis to randomisation was 15 days [14]. Median interval to neurologic progression was 10 months (delay 2 weeks or less) versus 8.8 months (delay 2–4 weeks) in the WBRT arm alone. Neither significance of this difference nor survival outcomes for different magnitudes of delay were reported. We were unable to identify more reports focussing on impact of waiting time. Danjoux et al. reported that initiation of a rapid response radiotherapy program was effective in reducing waiting time for palliative radiotherapy [15]. Impact of waiting time on survival was not analysed in detail. Whether or not waiting time compromises outcome might depend on primary tumour type, its biological behaviour, and related differences in growth rate or doubling time. In patients with nonsmall cell lung cancer waiting for stereotactic radiotherapy, only 23% of tumours did not show volume increase during waiting times >25 days [16]. In squamous cell head and neck cancer, 62% of cases had measurable increase in tumour volume before radiotherapy, median 46% (median interval between imaging studies 28 days) [17]. Other tumours might grow less rapidly. However, for individual patients prediction is often difficult if not impossible. Indirectly, longitudinal studies such as those in lung and head and neck cancer, provide arguments against waiting times in excess of 2-3 weeks. Regarding brain metastases, there is a need to perform further analyses, for example, based on published randomised studies that included prospective documentation of endpoints other than survival and larger groups of patients.

## Figures and Tables

**Table 1:** Patient characteristics (*n* = 110): no significant difference in age, KPS, number of brain metastases, extracranial metastases, primary tumour control, DS-GPA and RPA classification, radiation dose, and time interval (*P* = 0.22 or more); significant difference: primary disease type (*P* = 0.03).

Parameter	Interval < 12 days	Interval ≥ 12 days
Median age, years	64	64
Median KPS	60	70
Median DS-GPA score (min. 0, max. 4 points)	1.0	1.0
Number of BM: 1 (%)	5	9
Number of BM: 2-3 (%)	17	16
Number of BM: >3 (%)	25	27
Extracranial metastases (%)	43	38
No extracranial metastases (%)	5	15
Uncontrolled primary tumour (%)	16	25
Controlled primary tumour (%)	31	28
DS-GPA class I versus II versus III versus IV (%)	2, 4, 9, 33	1, 5, 17, 30
RPA class I versus II versus III (%)	2, 20, 25	3, 26, 24
NSCLC, SCLC (%)	15, 8	32, 2
Breast, MM, and GI (%)	9, 3, 6	3, 4, 6
Kidney, others (%)	4, 2	4, 3
Total dose 20 Gy (%)	12	17
Total dose 30 Gy (%)	35	35
Simultaneous detection of BM and primary tumour (%)	8	17
Time interval to BM 1–12 months (%)	23	17
Time interval to BM 13–36 months (%)	7	9
Time interval to BM > 36 months (%)	9	8

KPS: Karnofsky performance status; BM: brain metastases; DS-GPA: diagnosis-specific graded prognostic assessment; NSCLC: nonsmall cell lung cancer; SCLC: small cell lung cancer; MM: malignant melanoma; GI: gastrointestinal primary tumour.

**Table 2:** Median survival after whole-brain radiotherapy (WBRT). All *P* values are 0.29 or worse.

Group	Median survival from WBRT	Median survival from diagnosis
Interval < 12 days	2.6 months	2.8 months
Interval ≥ 12 days	2.5 months	3.3 months
Start within 1 week	2.3 months	2.4 months
Start during week 2	2.2 months	2.6 months
Start during week 3	2.7 months	3.3 months
Start after > 3 weeks	2.8 months	4.2 months
